# Molecular Mechanisms of Neonatal Brain Injury

**DOI:** 10.1155/2012/506320

**Published:** 2012-01-26

**Authors:** Claire Thornton, Catherine I. Rousset, Anton Kichev, Yasuka Miyakuni, Regina Vontell, Ana A. Baburamani, Bobbi Fleiss, Pierre Gressens, Henrik Hagberg

**Affiliations:** ^1^Centre for the Developing Brain, Institute of Reproductive and Developmental Biology, Department of Surgery and Cancer, Imperial College London, Hammersmith Campus, Du Cane Road, London W12 0NN, UK; ^2^Perinatal Center, Institutes of Clinical Sciences & Neuroscience and Physiology, The Sahlgrenska Academy, University of Gothenburg, 405 30 Gothenburg, Sweden; ^3^Inserm, U676, 75019 Paris, France; ^4^Faculté de Médecine, Université Paris Diderot, 75013 Paris, France

## Abstract

Fetal/neonatal brain injury is an important cause of neurological disability. Hypoxia-ischemia and excitotoxicity are considered important insults, and, in spite of their acute nature, brain injury develops over a protracted time period during the primary, secondary, and tertiary phases. The concept that most of the injury develops with a delay after the insult makes it possible to provide effective neuroprotective treatment after the insult. Indeed, hypothermia applied within 6 hours after birth in neonatal encephalopathy reduces neurological disability in clinical trials. In order to develop the next generation of treatment, we need to know more about the pathophysiological mechanism during the secondary and tertiary phases of injury. We review some of the critical molecular events related to mitochondrial dysfunction and apoptosis during the secondary phase and report some recent evidence that intervention may be feasible also days-weeks after the insult.

## 1. Introduction

Brain injury occurring during the perinatal period is a common cause of life-long neurological disability. The etiology is complex and multifactorial, but hypoxia-ischemia (HI), infection/inflammation, and excitotoxicity are considered important causes or precipitating insults of preventable/treatable forms of perinatal brain injury. Genetic background, maturational age, sex, and degree of brain development of particular regions affect vulnerability and the mechanisms of brain injury [1, 2]. Furthermore, antecedents like infection/inflammation, intrauterine growth restriction, or preexposure to hypoxia can modulate brain vulnerability [3–5]. Brain injury evolves over time, and different mechanisms are critical during the primary, secondary, and tertiary phases. Indeed, recent experimental data suggests that interventions can be effective if administered hours, days, or even weeks after the primary insult [6, 7].

The aim of the present paper is to describe the critical mechanisms of brain injury during the different stages after an acute insult with particular emphasis on mitochondrial impairment, apoptotic events and the tertiary phase of injury.

## 2. Secondary Brain Injury

Cerebral HI that is sufficiently severe to cause depletion of tissue energy reserves (primary insult) is often followed by transient but complete restoration of glucose utilization, ATP and phosphocreatine upon reoxygenation [8–10]. Thereafter a secondary decrease of high energy phosphates occurs in experimental studies that parallel a decrease in tissue glucose metabolism and development of cell injury [8–10]. In a similar way, infants with neonatal encephalopathy exhibit characteristic abnormalities in cerebral energy metabolism, which is frequently normal soon after birth, but shows a progressive decline in [PCr]/[Pi] some hours later [11]. Infants displaying this phenomenon develop neurodevelopmental impairment or die, and there is a close relationship between the magnitude of the late decline in [PCr]/[Pi] and the severity of long-term neurodevelopmental impairment [12].

These findings suggest that most of the injury after HI evolves with delayed onset *after *rather than during the insult. There are many examples of successful posttreatment after HI in animals suggesting a therapeutic window following HI prior to the secondary phase of tissue impairment [13]. Hypothermia following HI reduces secondary energy failure and brain injury in newborns with neonatal encephalopathy [14]. However, the mechanisms involved in secondary brain injury are largely unknown and such knowledge is critical for development of future therapies for preterm infants or to be combined with hypothermia in severely asphyxiated infants at term, hopefully, to further reduce serious disability in children and adults.

## 3. Mitochondrial Functional Impairment

Mitochondria are small membrane-enclosed organelles, remarkably mobile and plastic, constantly changing their shape and undergoing fusion and fission [15]. Many factors can challenge mitochondrial balance and good functioning: DNA mutations, increase of intracellular calcium, reactive oxygen species, inflammation, decrease in trophic factors, and mitochondrial dysfunction plays a crucial role in brain injury [16]. Because of the heterogeneity of mitochondria existing in the brain, to understand variations in mitochondria functioning and consequent selective vulnerability to injury, the organelle must be placed within the context of its cellular, functional, developmental, and neuroanatomical environment [17, 18]. The location of mitochondria in the cell varies between cell types, but they are most often localized near sites of high ATP utilization as their major role is to produce and supply energy, ATP, to the cells through the enzyme complexes forming the respiratory chain. Mitochondrial function is critically important during development and throughout life in metabolic tasks like cellular proliferation, regulation of the cellular red-ox state, apoptosis, and excitotoxic injury.

Interest is growing in mitochondrial diseases or mitochondria-related injury where the respiratory chain/oxidative phosphorylation system starts to malfunction. Mitochondrial diseases are principally due to mutations in either nuclear or mitochondrial DNA, provoking impairment of transcription, translation and assembly of the enzyme complexes, leading to the malformation and/or malfunction of the mitochondria [19, 20]. Impairment of the respiratory chain is associated with ageing, neurodegenerative disorders [21], and mitochondrial diseases [19]. During ageing, inefficiency of the respiratory chain has been linked to the decreased activity of AMP-activated protein kinase (AMPK) leading to decreased mitochondrial biogenesis and function [22, 23]. In neurodegenerative disorders, like Parkinson's and ALS, an increase of oxidative stress is shown to be a crucial initiator affecting respiratory chains, leading ultimately to cell death [21, 24]. As well, recent discoveries of mutation associated with hereditary form of those diseases render the story even more complex [25].

Very little is known of what happens to the respiratory chain in injuries like stroke or during perinatal brain damage. After neonatal hypoxia-ischemia (HI), there is a significant energy failure in the brain, followed by a recovery period before a second energy failure [2, 26–29]. Those primary and secondary energy failures are associated with the primary and secondary injury [30]. Currently, most of the research on perinatal brain damage is focusing on the secondary insult leading to cell death and tissue injury [31]. However, what is happening during the primary energy failure, what is happening during the short recovery, and what mechanisms lead to the second energy failure and injury remain unknown.

## 4. The Role of AMPK in Mitochondrial Energy Crisis

Challenges to mitochondrial biogenesis and integrity are most likely to happen quite early in the cascade of events leading ultimately to injury. Before being involved in the apoptotic process after HI ([31–33] and see paragraph below) and considering the role of mitochondria as a major ATP supplier, it is most likely that mitochondria are involved from the first steps of the injury process after the insult. For instance, our group recently identified a peak of AMPK activity as early as 20 min after an HI insult in the brain of neonatal mice (Rousset et al., unpublished data). AMPK is well known as the energy sensor of the cell and is activated when there is an imbalance in the AMP : ATP ratio such as that which occurs in heat shock, anoxia, and so forth [34]. Once activated, AMPK will inhibit energy-consuming pathways (fatty acid/cholesterol synthesis) and promote energy-producing pathways (glycolysis, e.g., or through PGC-1*α* increasing mitochondria biogenesis, [35, 36]) in an attempt to restore energy balance which is critical to cell survival. AMPK is activated through two upstream kinases: LKB1 and CaMKK*β* [37–41]. The latter is activated by a surge of intracellular calcium within the cell [40], which happens during excitotoxicity, a well-described feature of HI injury mechanism [42]. Furthermore, AMPK has recently been shown to mediate apoptosis through expression of the proapoptotic protein Bim after an excitotoxic challenge *in vitro* [43].

**Figure 1:**
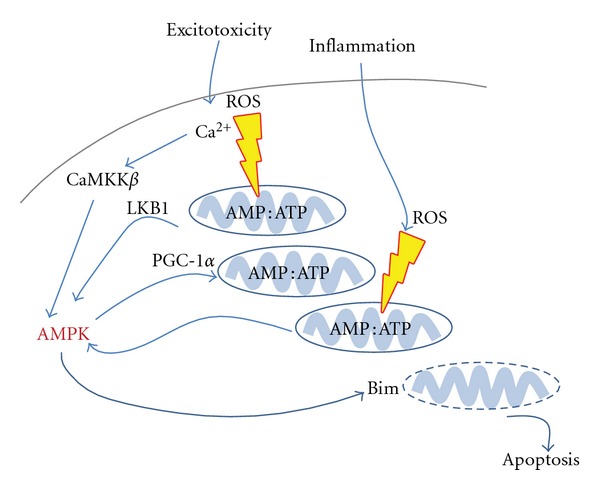
A potential role for AMPK in neonatal brain injury. AMPK is activated in response to stresses which change either intracellular calcium levels (e.g., excitotoxicity) or deplete intracellular ATP concentrations (e.g., inflammation, reactive oxygen species). Although AMPK works to return energy levels to baseline, prolonged activation results in upregulation of the proapoptotic protein, Bim.

Hypothetically, as a first step, the calcium surge provoked by excitotoxicity and ROS signalling [44, 45] could not only activate CaMKK*β* and then AMPK but could also simultaneously challenge the mitochondrial respiratory chain leading to an imbalance in the AMP/ATP ratio, reinforcing AMPK activation through the second upstream kinase LKB1. The activation of downstream pathways of AMPK to restore energy balance, could logically explain the return to basal level of ATP in the brain after the primary energy failure. Subsequently, events in the mechanistic cascade responsible for HI injury, like inflammation [32], could theoretically once again impede mitochondrial function, causing the secondary energy failure (Figure 1). This, cumulating with an overactivation of AMPK, which has been reported to exacerbate injury after stroke [46, 47], and still ongoing deleterious consequences from previous events, could provoke in the most vulnerable cells a final mitochondrial challenge, leading to its membrane permeabilisation and ultimately cell death through apoptotic pathways.

## 5. Mitochondrial Fusion and Fission

Mitochondria constantly fuse and divide, and the mechanisms governing this aspect of mitochondrial behaviour are currently the focus of many investigations. This property to fuse and divide appears to be crucial for a number of functions, the maintenance of organelle fidelity, mediating DNA or protein quality control, and, finally, it may be an important feature during apoptosis [48]. Mitochondrial fusion proteins attenuate apoptosis by inhibiting the release of proapoptotic agents like cytochrome c, while mitochondrial fission protein DRP-1 promotes apoptosis through Bax, leading to mitochondrial outer membrane permeabilization and cell death [49]. However, it is of note that fusion and fission have not yet been investigated in the immature brain, but this is surely something of great interest to push forward.

## 6. Intrinsic Pathway of Apoptosis and Secondary Brain Injury

Apoptosis (programmed cell death) is essential for the normal development of tissues and is especially key in neuronal development. The balance between cell survival and cell death is therefore required to be highly regulated; as such it is unsurprising that aberrant activation of apoptotic pathways occurs in a number of pathological conditions including stroke and a variety of neurodegenerative diseases [50].

Cellular apoptosis can be achieved through two routes, the extrinsic pathway (discussed later) activated in response to extracellular signals such as Fas and TNF*α* and mediated by death receptors [51] and the intrinsic pathway activated in response to DNA damage or cellular stress. Although each pathway has unique members, both mechanisms converge downstream at the level of the mitochondrion, where if the insult is severe enough, there is catastrophic permeabilisation from which the cell cannot recover. Mitochondrial permeabilisation results in the release of mitochondrial apoptogenic factors into the cytosol including apoptosis-inducing factor (AIF), endonuclease g (endo G) cytochrome c (cyt c), and Smac/Diablo. These proteins have a number of pro-apoptotic functions; cyt c interacts with Apaf-1 to form an active apoptosome, providing a platform for procaspase-9 cleavage; Smac/Diablo interacts with inhibitors of apoptosis (IAP) reducing their negative influence on the activity of caspases [50]. In contrast with cyt c and Smac/Diablo, AIF and endo G operate through a caspase-independent pathway. Both are translocated to the nucleus from the mitochondria in response to death—inducing stimuli where they induce fragmentation of nuclear DNA [52, 53].

## 7. The Role of Caspases in Neonatal Brain Injury

Caspases play a key role in apoptosis and inflammation. Caspases can be divided into three groups: initiator caspases (caspase-2, -8, -9, -10), effector caspases (caspase-3, -6, -7), and inflammatory caspases (caspase-1, -4, -5, -11, -12). Whereas effector caspases are activated by the initiator caspases, initiator caspases are activated by different, more complex mechanisms [54].

In the extrinsic pathway, binding ligands to death receptor leads to recruitment of adaptor protein, which recruits caspase-8, forming DISC (death-inducing signaling complex) leading to dimerization and activation of caspase-8. Caspase-8 then cleaves and activates effector caspases. In the intrinsic pathway, after cyt c is released from mitochondria into cytosol, it interacts with Apaf-1. This complex binds to procaspase-9 in the presence of dATP/ATP and forms the apoptosome which cleaves and activates initiator caspase, caspase-9 which, in turn, activates effector caspases (in particular, caspase-3) by cleaving between their large and small subunits [55]. Activated effector caspases cleave cellular substrates, such as PARP (poly(ADP-ribose) polymerase), lamin, fodrin, ROCK1 (Rho-associated kinase 1), and ICAD (inhibitor of CAD), leading to DNA fragmentation, cell shrinkage, and membrane blebbing [56–58]. Among the effector caspases, caspase-3 cleaves a broad range of substrates and the main effector caspase in the brain.

During brain development, a large number of neurons are eliminated by apoptosis to optimize neural networks. The activation of caspase-3 appears in the execution of neuronal apoptosis in the brain during development and after acute injury like HI. The extent of caspase-3 activation following brain injury is greater in immature brain than adults [59, 60]. Caspases are important for apoptosis in developing brain. Nevertheless, there is the implication that caspase-independent death pathways may also influence nervous system development and may provide an alternative mechanism for regulating neuronal death.

The initial report characterising caspase-3-deficient mice showed defects of apoptosis in the nervous system; these mice die during embryonic development or in the perinatal period, in a manner similar to the phenotype of caspase-9 and Apaf1-deficient mice. Subsequently, it was reported that caspase-3 deficiency on C57/BL/6J background produced only minor neuropathological changes and caspase-3-deficient C57/BL/6J mice survived into adulthood [61]. Moreover, neonatal HI brain injury in caspase-3-deficient mice is worse compared with the previous model [62]. In rats subjected to neonatal HI, there is a peak of caspase-3 activity observed 24 h after the insult which remains elevated for a significant number of days [63]. These data suggest that the apoptotic pathway is likely to be strain dependent and caspase-independent death pathways may also influence nervous system development and may provide an alternative mechanism for regulating neuronal death. Recent studies have also revealed the nonapoptotic function of caspases. In particular, caspase-3 is suggested to function in neurogenesis and synaptic activity [64].

Caspase-6 is an effector caspase, and, in apoptotic pathways, lamin, a structural protein of nuclear envelope, is thought to be the only substrate cleaved exclusively by caspase-6. In other pathways, caspase-6 is also known to cleave cytoskeletal and structural proteins, such as the microtubule-associated protein tau and amyloid precursor protein (APP), and caspase-6 is detected in neurodegenerative diseases, such as Alzheimer's disease and Huntington's disease. Recently, Nikolaev and colleagues identified APP/death receptor-6 (DR6)/caspase-6 pathway as the mechanism specific for axonal pruning and degeneration by trophic factor withdrawal in developing neurons [65]. As a result, the involvement of caspase-6 in axonal degeneration has come under a high degree of scrutiny [66, 67]. Recently, it was demonstrated that *caspase-6* gene deficiency conferred protection in a mouse model of adult stroke with a reduction of axonal degeneration and improvement of functional outcome [66]. We have recently found that caspase-6 is activated (cleaved) also in neurites in the immature brain after HI (Miyakuni et al., personal communication), but its pathophysiological importance remains unknown.

## 8. A Role for Mitochondrial Permeabilisation in Secondary Brain Injury in Neonatal HI

**Figure 2:**
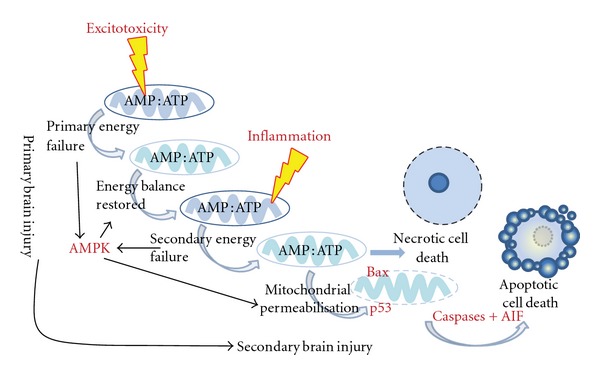
The development of secondary brain injury. Energy depletion culminating in Bax-dependent mitochondrial permeabilisation represents an irreversible commitment to cell death in neonatal brain injury.

Mitochondrial permeabilisation (MP) therefore represents the “point of no return” in the life cycle of the cell. Two forms of permeability have been identified. Mitochondrial outer membrane permeability (MOMP) is the result of Bcl-2 family members such as Bax relocating from the cytosol to the mitochondria. Once there, Bax interacts with another Bcl-2 family member Bak to form pores in the outer membrane enabling proteins located between the inner and outer membranes to leak into the cytosol [68]. In contrast, a permeability transition pore (PTP) is formed at points where both the inner and outer leaflets of the mitochondrion are at their closest points. In contrast with MOMP, the inner mitochondrial membrane is permeabilised resulting in leakage of solutes, depolarisation due to proton gradient equilibration, and generation of reaction oxygen species. ATP production ceases and the mitochondrion swells ultimately disrupting the outer membrane. PTP-mediated cell death is predominantly necrotic (through calcium imbalance and bioenergetic failure), although in extreme cases, if sufficient ATP is present, apoptosis can occur through activation of caspases [69]. Induction of the PTP is enhanced by cyclophilin D, a mitochondrial matrix protein which has previously been implicated in adult ischaemic injury [70]. However, our recent studies demonstrated that Bax-mediated MOMP rather than cyclophilin-D-mediated PTP is critical in mouse models of neonatal HI [71]. Indeed, previous work from our group and others suggests that, in neonatal brain, Bax-dependent mitochondrial outer membrane permeabilisation is implicated (Figure 2).

## 9. Involvement of Bax and Other Proapoptotic Bcl-2 Family Members in Neonatal HI

A study examining Bax-deficient mice found that these animals were protected in immature brain injury paradigms [72]. Furthermore, studies which ablate the effects of Bax-mediated mitochondrial membrane permeabilisation (e.g., knockout models of Bim and Bad [73], Tat-Bcl-xL-mediated neuroprotection [74], *Bcl-xL* transgenic mice [75]) all exhibit reduced brain injury after neonatal HI. Pharmacologically, intracerebroventricular injection of Bax inhibitory peptide prior to induction of HI in a neonatal mouse model conferred neuroprotection in both grey and white matters [76]. Finally, both caspase-dependent and AIF pathways are activated to a much greater extent in the immature brain compared with the adult brain [60]. Taken together, these data suggest that Bax-dependent mitochondrial permeabilisation is a critical event in delayed brain injury because it leads to both activation of caspase-dependent and caspase-independent cell death and mitochondrial functional impairment.

## 10. Upstream Regulators of Proapoptotic Bcl-2 Family Members

### 10.1. p53

It is a tumour suppressor that triggers apoptosis via multiple pathways including cell cycle arrest and the regulation of autophagy through transactivating pro-apoptotic and repressing antiapoptotic genes [77]. It is highly conserved and regulates cell death resulting from a wide variety of both physiological and pathological stimuli [78]. p53 also has transcription-independent, cytoplasmic actions at the mitochondrial level and can promote Bax-dependent mitochondrial permeabilisation [79]. In unstressed neurons, p53 expression is generally low, limited by its association with its negative regulator MDM2 which functions as a ubiquitin ligase, targeting polyubiquitinated p53 for degradation [80]. Cellular stress displaces p53 from MDM2, and subsequently p53 expression is stabilised through substantial posttranslational modification [77]. The classical role for p53 is as an activator of transcription, and, on stabilisation, it accumulates in the nucleus where it upregulates the transcription of proapoptotic genes such as *PUMA, BAX,* and *Noxa *[81]. More recently a cytosolic, transcription-independent role was described in which activated p53 accumulates in the cytosol where it is sequestered by the antiapoptotic Bcl2 proteins for example, Bcl-xL [79]. However, increased PUMA expression mediated by nuclear p53 displaces Bcl-xL allowing p53 to activate Bax, promoting its oligomerisation, mitochondrial outer membrane permeabilisation, and inducing apoptosis [79, 82].

A previous study found that p53 was upregulated and accumulated in the nucleus and mitochondria in an *in vivo* rat model of neonatal HI. In consequence, there was an upregulation of apoptotic pathways leading to activation of caspase-3. The authors identified a pathway involving NF*κ*B upstream of p53 and were able to decrease p53 accumulation (thus increasing neuronal survival), in response to neonatal HI by treating with the NF*κ*B inhibitor NBD peptide [83, 84]. Subsequently, this has translated into improved long-term function in behavioural tests [85]. More recently, the same group confirmed the importance of p53 activation in neonatal HI by use of a small molecule inhibitor of p53, pifithrin-*μ*. Injection of this peptide into mice which have previously been subjected to an HI paradigm results in a high degree of protection in both white and grey matters which translates into long-lasting behavioural benefits compared with sham-injected animals [86]. As pifithrin-*μ* is widely believed to inhibit the mitochondrial but not nuclear functions of p53 [87], this strengthens the case for critical involvement of a p53-Bax pathway in neonatal HI.

### 10.2. C-Jun N-Terminal Kinases (JNKs)

These are members of the mitogen-activated protein kinase (MAPK) family and, as such, are activated in response to stress. There are three mammalian *jnk* genes and 10 expressed isoforms as the result of alternative splicing; however, it is JNK3 that is predominantly active in the brain [88]. In a mouse model in which JNK3 expression is ablated (JNK3 KO), both adult and neonatal animals were partially protected against HI insult, and, in newborn animals, levels of c-jun were reduced compared with wild-type animals [89, 90]. This correlates with an earlier study suggesting that expression of *c-Jun* and its subsequent phosphorylation was increased on ischaemic injury [91]. JNK3 is hypothesised to act upstream of the proapoptotic Bcl-2 family as JNK3-mediated increases in Bim and PUMA expression were absent in the JNK3 KO animal [90]. In addition, activation of caspase-3 was also decreased suggesting that activation of JNK3 in response to hypoxic-ischaemic insult results in caspase-dependent apoptosis.

### 10.3. Caspase-2

It is a member of the initiator subgroup of caspases and is developmentally regulated [92]. Activation of caspase-2 is dependent on its dimerisation and subsequent cleavage which is facilitated through interaction with PIDD (p53-induced death domain-containing protein) and RAIDD (RIP-associated ICH-1/CED3 homologous protein with a death domain) [93–95]. Once activated, caspase-2 promotes Bid cleavage resulting in Bax translocation and release of cyt c [96]. In a very recent study, caspase-2 null newborn mice were found to be partially protected in both excitotoxic and HI paradigms [97] in contrast with the adult caspase knockout mouse model [98]. As the study also showed high expression of caspase-2 in neonatal mice and rats which decreased postnatally, it is probably unsurprising that there are age-dependent differences in caspase-2 function. Interestingly, a group II caspase inhibitor, TRP601, has recently been developed which targets caspase-2 and caspase-3 functions. Neonatal animals subjected to excitotoxicity, arterial stroke, or HI insult were significantly protected against white and grey matter loss [99].

## 11. Death Receptors and the Extrinsic Pathway of Apoptosis

During inflammation such as that which has been reported in perinatal brain injury [32], activation of mast cells [100] and microglia will produce reactive oxygen species, release excitatory amino acid agonists, proinflammatory cytokines (e.g., IL-1*γ*, IL-18, TNF-*α*), chemokines [101, 102], and tumour necrosis factors (e.g., TNF-*α*, TNF-*β*, FasL, TRAIL, TWEAK) [101, 103–105] that will contribute to cell death most often characterized by a mixed apoptotic-necrotic phenotype [59, 106].

**Figure 3:**
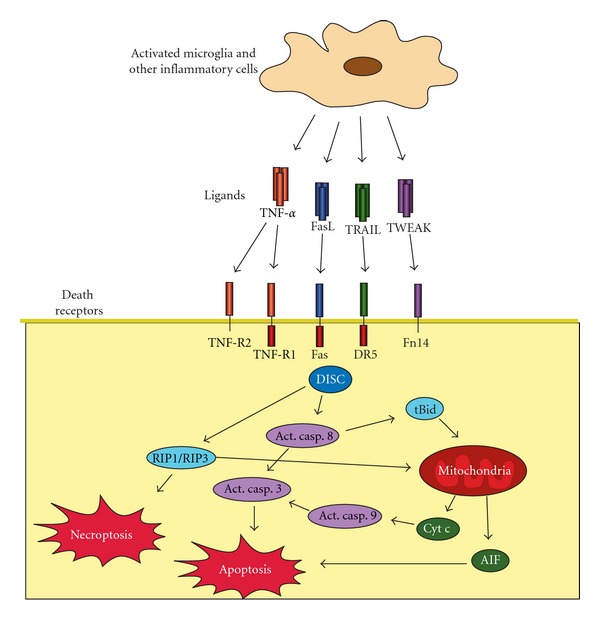
The extrinsic pathway of apoptosis. Inflammatory cells secrete death receptor ligands which bind to receptors on neurons, oligodendroglial and other receptor-expressing cells, recruiting the death-inducing signalling complex (DISC) and triggering both apoptotic and necroptotic pathways.

From the time TNF was cloned and characterized in 1984 [107], roughly 20 ligand-receptor pairings are now included in the TNF superfamily. These TNF and TNF-receptor-like molecules are similar in structure to TNF and are functioning as trimers (both ligands and receptors). The receptors are largely membrane-bound signalling molecules with exception of some soluble decoy receptors (e.g., Osteoprotegerin). The ligands instead can be either membrane or soluble forms and both forms can have physiological activity. Because of the similarity of their structure, multiple ligands are able to bind and induce signalling through one receptor, or a single ligand is able to bind multiple receptors. Some of the receptors contain the so-called death domain in their intracellular domain (e.g., TNF-R1, DR4, DR5, Fas) and are able to trigger apoptosis when activated from the binding of the corresponding ligand (e.g., TNF- *α*, TRAIL, FasL). This extrinsic pathway of apoptosis continues with the activation of a death-inducing signalling complex (DISC) adjacent to the death domain of the receptor. Activated DISC catalyzes the proteolytic cleavage and transactivation of procaspase-8 [108]. Activated caspase-8 either directly activates caspase-3 or mediates cleavage of Bcl-2 interacting domain (Bid) to truncated Bid (tBid), which integrates different death pathways at the mitochondria ([109]; Figure 3). tBid translocates to mitochondria where it interacts with other proapoptotic proteins and triggers the release of apoptogenic factors like cyt c and apoptosis-inducing factor (AIF) from the mitochondria. Apoptosis then proceeds in the same way as for the intrinsic pathway with caspase-dependent and caspase-independent cell death.

## 12. Necroptotic Cell Death

Activation of death receptors in the presence of broad-spectrum caspase inhibitors induces a newly described cell death process called necroptosis. Necroptotic cell death initiated by TNF-*α*, Fas, or TRAIL is mediated by formation of a complex of two kinases, RIP1 and RIP3. This complex promotes mitochondrial reactive oxygen species (ROS) production and eventual collapse of cellular energy production [110].

## 13. Involvement of Death Receptors in Neonatal Brain Injury

TNF-*α* activity is mediated through activation of two receptors: low, affinity TNFR1 (p55) and the high-affinity TNFR2 (p75) [111], found on both neuronal [112, 113] and glial cell populations [114]. Although the extracellular domains of both receptors have a high degree of homology, their intracellular domains differ significantly [115]. This leads to complex signal transduction pathways that can be triggered and may result in activation of the antagonistic functions of these two receptors [111, 116]. When activated, the intracellular part of TNFR1 containing the death domain triggers apoptosis [117], whereas TNFR2 lacks that domain—its activation triggers neuroprotection through activation of NF*κ*B [118]. There are several pieces of evidence that suggest the involvement of the TNF pathway in the development of white matter damage (WMD). Children who develop cerebral palsy show increased blood levels of TNF-*α* [119], and TNF receptor 1 is critical for LPS-mediated sensitization to oxygen glucose deprivation *in vitro* [120]. Moreover, deletion of the TNF gene cluster abolishes LPS-mediated sensitization of the neonatal brain to HI insult [121]. TNF-*α* treatment appears to be toxic for the oligodendroprecursor (OPC) cell [122] and potentiates the IFN-*γ* toxicity on those cells *in vitro* [123]. TNF-*α* has also been shown to stimulate astrocyte [124] and microglial [114] activation and proliferation. TNF-*α*-mediated cell destruction may be mediated directly, via activation of its TNFR and subsequent cell death signalling pathways, or indirectly by enhancing glutamate excitotoxicity [125]. TNF is also implicated in brain neuroprotection. It is shown that neuronal damage by focal cerebral ischemia and excitotoxic insults are enhanced in TNFR KO mice [126]. The neuroprotective role for TNF in cerebral ischemia is mainly attributed to TNFR2 activity [127].

FasL is able to bind with Fas death receptor triggering apoptosis and with Decoy receptor 3 (DcR3) [128]. Fas death receptor is one of the most extensively studied of this group of receptors. Lack of functional Fas receptor is neuroprotective in adult models of HI [129, 130]. HI also activates Fas death receptor signalling in the neonatal brain especially in areas where apoptosis is a prominent feature [131–133]. Although the Fas/FasL system is primarily linked to apoptosis, Fas activation can also induce caspase-independent cell death [134], initiate cell necrosis [135], or induce proliferation and differentiation signals [136]. It is shown that Fas expression in primary OPC is higher than in mature oligodendrocytes [123], implying higher susceptibility to FasL at earlier developmental stages. Fas expression can be upregulated in OPCs exposed to an inflammatory stimulus [123] which may imply that in an inflammatory environment these cells would have increased vulnerability to Fas-induced apoptosis.

In humans, four membrane-bound and one soluble receptor for TRAIL have been identified. Of these, two contain cytoplasmic death domain (DR4 and DR5) and have the capacity to induce apoptotic cell death [137, 138], whereas DcR1 (TRAIL-R3) and DcR2 (TRAIL-R4) lack functional death domains and thus are considered to act as decoy receptors [139, 140]. Osteoprotegerin (OPG) is a secreted TNF receptor family member that besides receptor activator of nuclear factor kappa-B ligand (RANKL) can bind TRAIL as well [141, 142]. In mice, two membrane decoy receptors mDcTRAILR1 and mDcTRAILR2 have been reported [143], one soluble OPG [142], and only one death-mediating TRAIL receptor which has the highest homology with the human TRAIL receptor DR5 [144].

Only one receptor for TWEAK has been identified so far in both humans and rodents, fibroblast growth factor-inducible 14 (Fn14) [145]. Binding of TWEAK to this receptor can trigger proliferation, differentiation, migration, and cell death [146]. The Fn14 cytoplasmic tail does not contain a canonical death domain, and TWEAK binding to Fn14 can induce multiple cell death pathways in different cellular contexts [147, 148].

Although many studies have been conducted in the cancer- or inflammation-related systems, the role of TRAIL and TWEAK in the development of WMD after HI is still unclear. The studies that implicate TRAIL and TWEAK signalling in the pathogenesis of ischemic cerebral damage are performed in adult models of stroke or multiple sclerosis and concern mainly neurons [105, 148–150]. To date very few studies relate these pathways to OPC death [61]. However, intracerebroventricular injection of soluble DR5 receptor [150] or Fn14 [105] is able to reduce significantly the infarct volume after HI in adult rodent models, strongly implicating TRAIL and TWEAK signalling in neuronal cell death after HI.

## 14. Tertiary Brain Injury

Tertiary brain injury will be defined as that occurring following the commonly defined events of primary and secondary cell death. As outlined previously, perinatal brain injury is predominantly caused by inflammation/infection and hypoxic-ischemic events that cause metabolic dysfunction and cell death. Even after secondary cell death has subsided, effects on the brain persist including sensitisation to inflammation or injury, increased seizure susceptibility, impaired oligodendrocyte maturation and myelination, and persistent inflammation and gliosis [151–156]. More speculatively, perinatal inflammation is suggested to play a critical role in the pathogenesis of autism and schizophrenia [157–159].

When considering treatments for tertiary brain injuries, we could distinguish between strategies aiming at extending the window of therapeutic intervention from the acute phase to the subacute phase and strategies targeting more long-term events such as chronic inflammation or postlesional plasticity.

## 15. Extending the Window

One key issue for protecting the perinatal brain is the available window for intervention in the processes leading to cell death. From a clinical point of view, the longer this window, better the chance to implement viable interventions. For example, hypothermia has to be initiated within the first 6 hours of life to be protective in term infants with neonatal encephalopathy [160]. Such a short window does not allow applying this treatment to all neonates who might benefit from it. As a strategy to enhance the efficacy of hypothermia, some groups have been trying to extend the window of intervention of hypothermia by giving first an antiepileptic drug prior to delayed hypothermia. Using the classical Rice-Vannucci P7 rat model, Liu and colleagues have shown that a combination of low-dose topiramate administered 15 minutes after the HI insult and 3-hour hypothermia initiated 3 hours after the insult was neuroprotective while topiramate alone or hypothermia alone had no significant effect [161]. More recently, the same group showed that early administration of Phenobarbital also enhanced the efficacy of delayed hypothermia [162]. It remains to be seen if drugs used successfully in parallel with hypothermia, such as melatonin and xenon, might also be able to extend the therapeutic window of this treatment [163, 164].

An alternative strategy would be to use early but short-term hypothermia to enhance the window of opportunity for a protective drug. This strategy could allow reducing the duration of hypothermia. Accordingly, it was shown that fructose-1,6-biphosphate (FBP) was neuroprotective against neonatal excitotoxic cortical damage [165]. However, the drug had to be given within the first 8 hours to be neuroprotective. Interestingly, a moderate but transient (4 hours) cooling immediately after the insult extended the therapeutic window for FBP, as FBP administered 24 h after the excitotoxic insult was still significantly neuroprotective in these pups.

## 16. Targeting the Long-Lasting Inflammation

A recent and intriguing study performed in preterm infants with cerebral palsy [155] suggests that, at least in some patients with perinatal brain damage, there could be a long-lasting inflammation as measured by increased TNF-*α* levels in the plasma and the supernatants of peripheral blood mononuclear cells after lipopolysaccharide stimulation. This long-lasting altered inflammatory response could have deleterious effects on the progression of disease and/or on the clinical symptoms. If such a pathophysiological event was confirmed, recognizing and blocking such a persistent inflammation could be of therapeutic value.

Additional studies are necessary to confirm these new hypotheses and to determine whether or not there is a long-lasting CNS inflammatory process. Techniques such as PET with markers of microglia or MRI using ferromagnetic particles taken up by activated microglia could be instrumental in this perspective. Indeed, a study using this approach has revealed that for many years after traumatic brain injury in human adults microglia remain activated [166]. Although these studies have not yet been reproduced in children/young adults following perinatal injury, a similar activation might be ongoing and therefore a target for reducing tertiary phase injury.

## 17. Targeting Epigenetic Marks

The term epigenetics refers to the enzymatic (e.g., acetylation, methylation) and nonenzymatic mechanisms (microRNA) by which gene expression/cell phenotype is modified without altering the sequence of genomic DNA. Inflammation, growth restriction, and maternal stress are known to alter the epigenome [167–170], and although in the perinatal period these effects alone may not lead to classic brain injury, they may cause long-lasting cognitive, motor, and/or behavioural impairments [151, 167, 171].

The underlying mechanisms by which modifying the epigenome could have lasting effects includes myelin deficit linked to blockade of oligodendrocyte maturation, impaired neuronal migration, increased neuronal cell death, impaired axonal growth, or altered synaptogenesis [172–175]. Of particular interest, microRNAs with suggested roles in regeneration and repair are upregulated from 3 days after MCAO [176], and microRNAs are capable of enhancing the beneficial microglial M2 phenotype [177]. If microRNAs do indeed represent an endogenous repair and immunomodulatory mechanism, they may be a novel strategy to treat brain injury in the tertiary phase.

Drugs specifically targeting acetylation have shown great efficacy in treating acute-phase adult cerebral injuries (see, [178]), and evidence is mounting to suggest efficacy in neonatal models ([179]; Fleiss and Mallard, unpublished). We do not yet know if modulating the epigenome after the secondary phase will have any efficacy after inflammation or HI. However, adult changes in behaviour stemming from perinatal maternal stress and linked to increased methylation can be abolished in adulthood by increasing acetylation [180]. This raises hope for the future design of innovative treatments that could be implemented way beyond the perinatal insult.

## 18. Promoting Positive Post-Lesional Brain Regeneration with M2 Microglia

Activated microglia have been shown to be detrimental for the production of hippocampal neurons, but microglia and macrophages can also be beneficial and support neurogenesis, progenitor proliferation, survival, migration, and differentiation in other brain regions. Recent studies suggest that the phenotypic expression of macrophages can vary depending on the situation and pro-inflammatory macrophages (M1) can undergo transition into an anti-inflammatory-reparative (M2) phenotype. More recently, three activation states of microglia in CNS have been proposed: classical activation (tissue defence, pro-inflammatory), alternative activation (repair, anti-inflammatory, fibrosis, extracellular matrix reconstruction), and acquired deactivation (immunosuppression, phagocytosis of apoptotic cells [181, 182]).

Strategies aiming at activating microglia when it has reached the M2 phase could be beneficial for facilitating repair and plasticity. Of note, the early phases of microglial activation (M1 type of activation) have typically been described as deleterious for the brain. More recently, preventing early microglial activation has been shown to be detrimental in focal ischaemia [183, 184]. This suggests caution in timing of any intervention to modify microglial activity.

Alternatively, or in parallel, strategies aiming at accelerating the M1-M2 switch could also be of major interest. At this point, it is not known if modulation of the activation state of microglia/macrophages can be used for development of novel therapeutic strategies in the developing brain, but a recent report suggests that M2 (alternative activation/acquired deactivation) macrophage cell therapy indeed can provide protective effects in an animal model of multiple sclerosis [185].

## 19. Promoting Positive Post-Lesional Brain Regeneration with Exogenous Stem Cells

The development of an adequate protocol for stem cell culturing and application has envisaged the use of these cells for the reparation of perinatal cerebral lesions. Some studies have shown a positive effect of neural or mesenchymal stem cell therapy on the lesion extent and/or cognitive or motor outcome following perinatal brain lesions [7, 186]. Interestingly, in some of these studies, positive effects were observed when stem cells were injected several days (up to 10 days) after the insult. Furthermore, in an adult MCAO model, stem cells given even 30 d post-insult improved neurobehavioural scoring assessed 50 d later suggesting efficacy may be possible even in the tertiary phase of perinatal brain injury [187].

The therapeutic potential of neural stem cells in acute neonatal brain injuries has been evaluated in a rodent excitotoxic model [186]. Early (4-hour) and late (72-hour) neural stem cells implantation significantly reduced brain lesion size in this neonatal model. The implanted cells, modified *in vitro* prior to transplantation toward the oligodendrocytic lineage, were capable of migrating toward the lesion site even when implanted contralaterally to the lesion. At the lesion site, the neural stem cells underwent transient differentiation into neurons and oligodendrocytes but not astrocytes, suggesting that fate specification was achieved by the culture conditions. Pre-implantation cell fate determination may offer some ability to specifically target white matter injury, such as predominates in the injured immature brain [188–191]. In parallel with the reduction in lesion size, the injured mice displayed a persistent and marked improvement in temporal and spatial memory at 3 and 6 weeks of age compared to littermates given intracerebroventricular injections of saline or fibroblasts.

Similarly, it was recently shown that two administrations of bone marrow-derived mesenchymal stem cells to neonatal mice 3 and 10 days after unilateral right carotid artery occlusion on P9 produced a 46% improvement in sensorimotor function as observed in the cylinder rearing test and a 60% decrease in neuronal loss, compared with vehicle-treated animals [7]. Moreover, cellular proliferation and differentiation of the proliferated cells into cells expressing neuronal, oligodendroglial and astrocyte markers was observed. Interestingly, remodeling of the corticospinal tract correlated with sensorimotor improvement.

It is not clear yet whether the stem cells themselves or factors secreted by stem cells mediate the positive effect. Increased neurotrophin production with eventual loss of injected cells is linked to improvements [186], while in some studies cells become functionally integrated [192]. The ethical problem associated with the use of human stem cells is less evident in mesenchymal stem cells or stem cells derived from cord blood. Such cells permit an autologous transplant and do not entail the problem of immune tolerance of the transplanted cells. A clinical study is currently being performed using stem cells in children with neonatal encephalopathy at the Duke University [193].

A further intriguing alternative to treatment with stem cells is to stimulate the production of endogenous neuronal stem cells. It has already been shown that stem cells accumulate in the subventricular zone following an acute brain lesion. These results open a new perspective: the stimulation of this stem cell population to support the physiological reparation processes of a lesion. A variant of this strategy would be to redirect new cell production from astroglia to oligodendrocytes and neurons [194]. Critically, stem cell therapies and stimulating endogenous proliferation bears the theoretical risk of cancer induction [193].

## 20. Promoting Positive Post-Lesional Brain Regeneration with Pharmacological Agents

Fostering positive post-lesional plasticity appears a very promising strategy for delayed interventions aiming at improving long-term neurological and cognitive function. However, there is still limited knowledge about the cellular and molecular mechanisms underlying post-lesional brain plasticity.

Different growth factors, such as brain-derived neurotrophic factor (BDNF), nerve growth factor (NGF), insulin-like growth factor-1 (IGF-1), erythropoietin (EPO), or vasoactive intestinal peptide (VIP), have been shown to reduce delayed neuronal death in various animal models of perinatal brain damage [195–199]. As for hypothermia, the window for intervention, when tested, was rather restricted to the first hours after the insult. However, beyond their potential capability to prevent neuronal cell death, growth factors appear as good candidates to target mechanisms involved in plasticity such as proliferation of neuronal precursors, axonal growth and sprouting, or synaptogenesis and synaptic stabilization.

Accordingly, BDNF and VIP have been shown to promote axonal sprouting following excitotoxic injury of the periventricular white matter in newborn mice [198, 199]. Although growth factors like BDNF are big molecules unlikely to cross easily through the intact blood-brain barrier, ampakines, allosteric positive modulators of glutamatergic AMPA receptors, are small and diffusible molecules able to induce BDNF production in the brain when administered systemically. Interestingly, ampakines have been shown to mimic BDNF effects on axonal sprouting in the mouse model of excitotoxic white matter injury [200].

Similarly, melatonin was shown to promote plasticity using the same model of neonatal excitotoxic white matter damage [42]. Although melatonin did not prevent the initial appearance of white matter damage, it promoted repair of secondary lesion with axonal regrowth and/or sprouting. Recent data have shown that the window for intervention is at least 24 hours after the insult (Gressens P, personal communication). Behavioural studies support the hypothesis that melatonin-induced white matter histological repair is accompanied by improved learning capabilities. Neuroprotective properties of melatonin have been confirmed in several animal models of perinatal brain damage, including fetal sheep [201]. Melatonin is a safe compound, including newborns [202], and it crosses the blood-brain barrier as well as the placenta. Based on these data, a clinical trial testing the neuroprotective effects of melatonin has been initiated in preterm infants at high risk of developing brain damage and neurological handicap [203].

Although this study needs to be replicated, an intriguing clinical study has recently shown that EPO, when given on an average of 24 hours after birth, had very significant neuroprotective effects in human term infants with neonatal encephalopathy [204]. Evidently, the precise mechanism for this neuroprotection is unknown, but the timing of intervention argues on favour of an effect of EPO on post-lesional plasticity although a direct effect on delayed neuronal cell death cannot be excluded.

##  Authors Contribution

C. Thornton and C. I. Rousset contributed equally to this work. P. Gressens and H. Hagberg shared senior authorship.
